# Trends and bibliometric analysis on pediatric anesthesia from 2002 to 2022: A review

**DOI:** 10.1097/MD.0000000000035626

**Published:** 2023-10-27

**Authors:** Dijiao Ruan, Xu Tang, Xiaoli Li, Lianlian Li, Jing Hua

**Affiliations:** a Department of Anesthesiology, Banan Hospital of Chongqing Medical University, Chongqing, China; b Department of Anesthesiology, People’s Hospital of Chongqing Banan District, Chongqing, China.

**Keywords:** anesthesia, bibliometric, pediatric, research, trends

## Abstract

Pediatric anesthesia is one of the most concerning topics in our society. However, there is still a lack of a comprehensive overview of the research base and of future trends. This study aimed to guide beginners quickly learn the academic research on pediatric anesthesia and do their own studies by analyzing the articles of this field in the latest 21 years through bibliometric analysis. Literature scanning was conducted with the Web of Science database. Microsoft Excel, SPSS, VOSviewer, and CiteSpace were in this review. There was an increasing trend of articles on pediatric anesthesia, based on the analysis of 11,591 included articles. The top 3 most productive countries were the United States of America (4538), Canada (730) and Turkey (688). The most productive institutions were Boston Childrens hospital, Childrens Hospital Philadelphia and Ohio State University. Tobias, Joseph D (141), Kim, Hee-Soo (40) and Curley, Martha A Q (38) were the most active authors. Habre W (2017), Gross JB (2002) and Cravero JP (2009) are the articles cited more than 100 times during the analysis years. Anesthesia and Analgesia, Anesthesiology, Pediatric Anesthesia, were the core journals in this field. Cohort, simulation, sleep, postoperative complication are strongest burst keywords in recent years. This article summarizes the authoritative institutions, authors, literatures and frontier hotspots on pediatric anesthesia. Itwill be a valuable literature review and help beginners to quickly get started in the field, reduce unnecessary clueless and aimless learning, and greatly improve learning efficiency.

## 1. Introduction

Anesthesia is defined as using drugs or other methods to make the patients loss of physical sensation, with or without loss of consciousness, in order to achieve the purpose of painless, so as to meet the further surgery, examination, treatment and other traumatic exercise.^[1,2]^ Because of the different anatomy and physiology in children, their anesthesia management is unique.^[3]^ Although today anesthetic drugs and techniques can meet the needs of surgical anesthesia, there is still some problems like delirium and anxiety.^[4,5]^ In order to better understand the impact of anesthesia on children and explore better drugs for children, people pay attention to the anesthesia of children is increasing. The trend can be found in this article.

Bibliometric analysis is a subject using mathematical and statistical methods to conduct the quantitative analysis of all knowledge carriers like articles and book.^[6–8]^ Bibliometrics analysis can identify regional, institutional, or authorial partnerships between publications in a field of study and predict their potential development relationships.^[9–11]^ Co-Author analysis can find out which authors are active in a certain field and which period of time published the most articles.^[12,13]^ Citation analysis may reveal which articles, authors, institutions, and countries are more influential in a certain field.^[14,15]^ In recent years, with the development of the medical industry, more and more researchers and doctors use bibliometric analysis to find research hot spots and inspirations.^[7,16–18]^ The authors of literature review like bibliometric analysis come from all over the world and published their work in journals worldwide. Some of these works were even published in excellent journals in related fields.^[19–21]^

Although pediatric anesthesia has received increasing attention in recent years, there has been no comprehensive bibliometrics study of this topic. The purpose of this study was to exert bibliometric analysis to analyze the articles on pediatric anesthesia published from 2002 to 2022 to determine which countries, organizations and authors published more. Keywords and the most cited articles and journals in this field were included analysis, so as to reveal the research trends in this field, the most influential articles and the most productive countries and regions.

## 2. Materials and Methods

The literature review was conducted using the Web of Science (WoS) database including SCI-EXPANDED, SSCI, AHCI, CPCI-S, CPCI-SSH, ESCI, BKCI-S, BKCI-SSH. All articles containing the following search conditions: keywords in the “Theme” section with “pediatric anesthesia” or “pediatric stomatology anesthesia” or “pediatric sedation” or “children anesthesia” not “animal” in the “Theme” section between 2002 and 2022 had bibliometric analysis performed. Because these data were derived from big data analysis, ethical review is not necessary. Citespace 6.1.R6 software (https://citespace.podia.com/) and VOSviewer version 1.6.16 (https://www.vosviewer.com/) were used for bibliometric network visualizations.

Statistical analysis was performed using the SPSS (version26.0, IBM Corp, Armonk, NY) packages. Linear regression analysis was used to estimate the number of publications in the coming 4 years. Statistical data according to the rounding principle to take integers. *P* < .05 was considered significant.

## 3. Results

**Figure 1.**
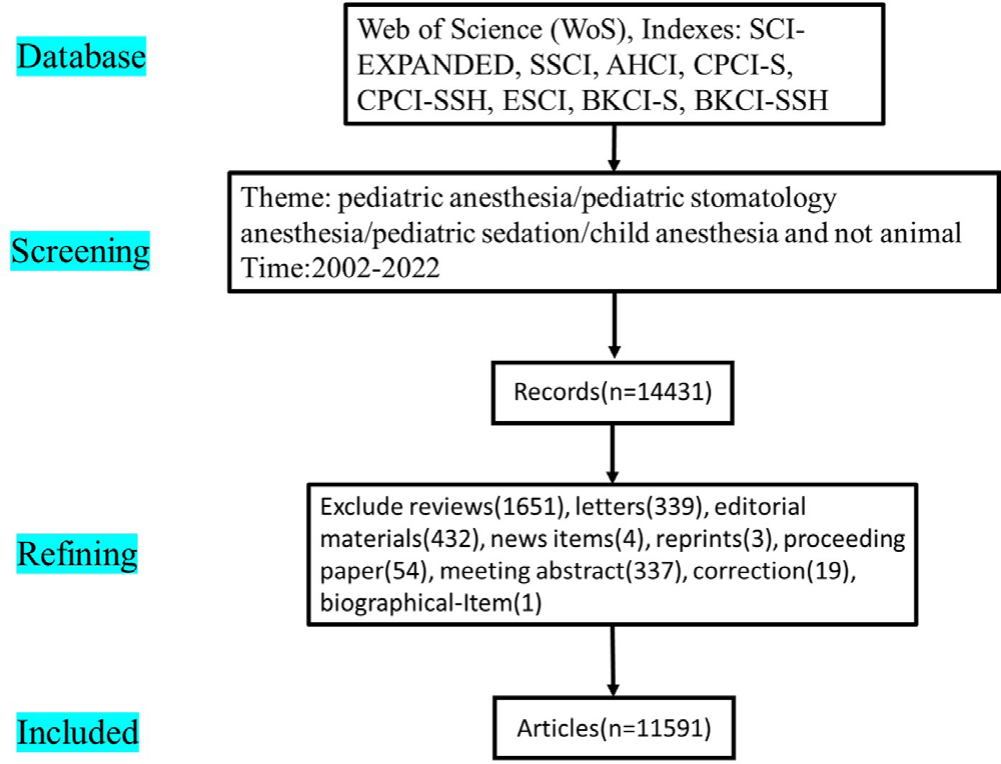
Data selection of this study for pediatric anesthesia.

According to the data select processing that show in Figure 1, a total of 14,431 records were found. Of these records, articles were 11,591, reviews were 1651, editorial materials were 432, letters were 339, meeting abstract were 337, proceeding paper were 54, correction were 19, news items were 4, reprints were 3, biographical-Item was 1. All the articles were included in our study to do the literature bibliometric analysis.

### 3.1. Trend of annual articles

**Figure 2.**
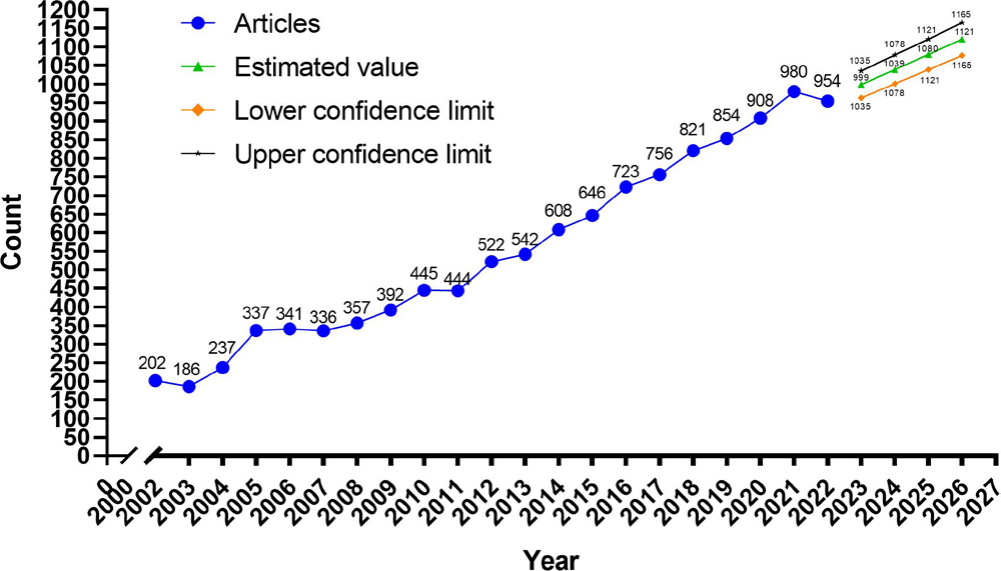
Annual trend articles of pediatric anesthesia.

Eleven thousand five hundred ninety-one unique articles were used to bibliometric analysis. The trend of annual articles was shown in Figure 2. It shows a significant linear growth trend in the articles on pediatric anesthesia per year over the past 21 years. And the number of published articles in the next 4 years were calculated using linear regression showing in the right of Figure 2 (Y = 40.63*year-81,190.12, R^2^ = 0.979). It estimated the mean number of published articles were 999 for 2023, 1039 for 2024, 1080 for 2025, and 1121 for 2026.

### 3.2. Productive countries and regions

**Figure 3.**
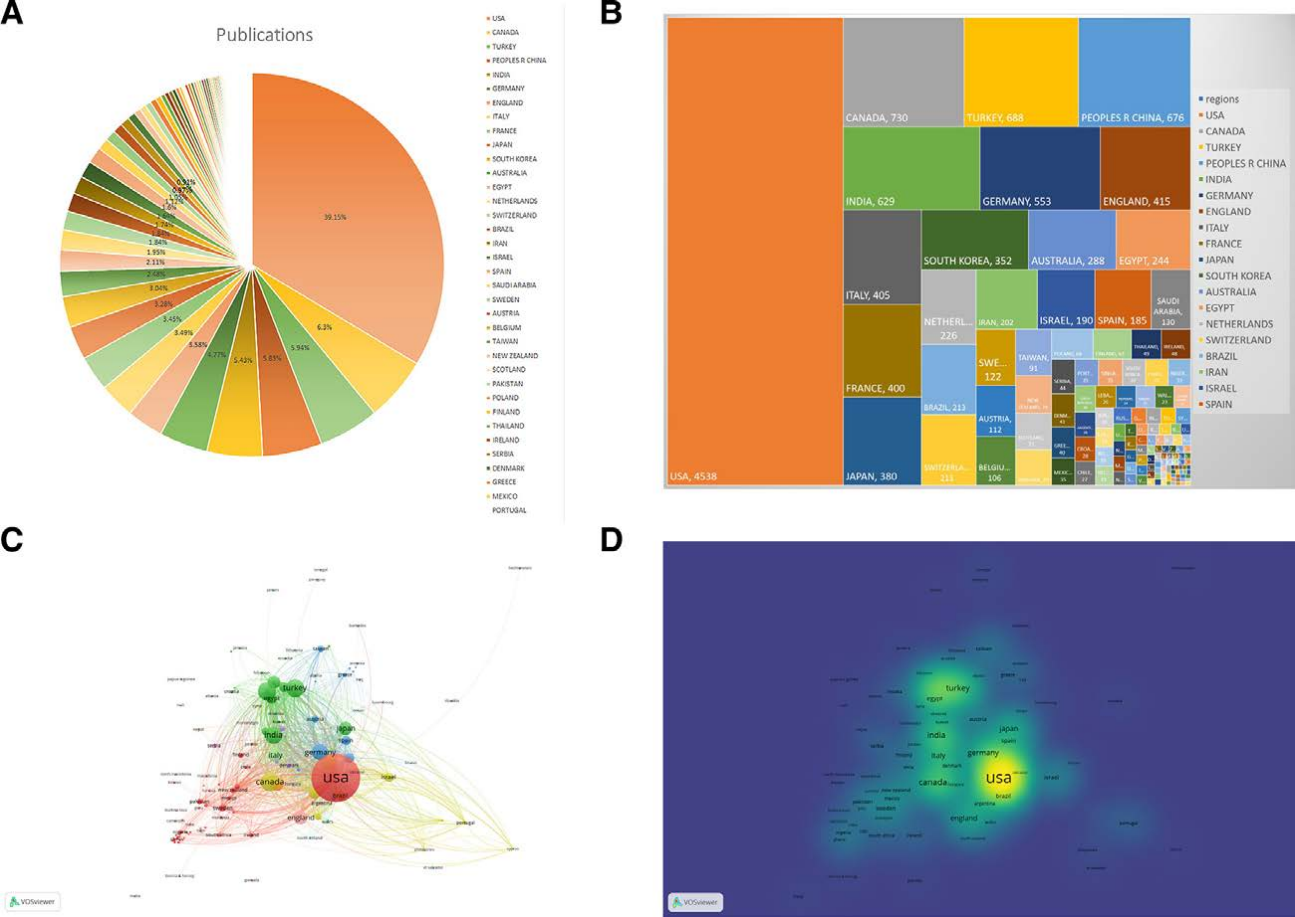
Countries/regions on pediatric anesthesia. Pie chart (A), treemap (B), network visualization map (C) and density map (D) of the articles published by country or region on pediatric anesthesia. (VOSviewer version 1.6.16).

The number of articles output by country and region is shown in Figure 3. A total of 136 countries output 11,591 papers. In this domain, USA had published the most publications with 4538 articles, followed by the Canada (730), the Turkey (688) and China (676). There were 23 active countries/regions produced more than 100 articles (Table 1). A pie chart, treemap and density visualization of published articles on pediatric anesthesia per country/region were shown in Figure 3A, B and D. A network map of country/region was displayed in Figure 3C. From this picture, we can find productive countries collaborated with each other very closely.

**Table 1:** The productive countries/regions with more than 100 articles from 2002 to 2022 on pediatric anesthesia.

Ranking	Countries	Publications	n % of 11,591
1	USA	4538	39.15
2	Canada	730	6.30
3	Turkey	688	5.94
4	Peoples R China	676	5.83
5	India	629	5.43
6	Germany	553	4.77
7	England	415	3.58
8	Italy	405	3.49
9	France	400	3.45
10	Japan	380	3.28
11	South Korea	352	3.04
12	Australia	288	2.48
13	Egypt	244	2.11
14	Netherlands	226	1.95
15	Switzerland	213	1.84
16	Brazil	213	1.84
17	Iran	202	1.74
18	Israel	190	1.64
19	Spain	185	1.6
20	Saudi Arabia	130	1.12
21	Sweden	122	1.05
22	Austria	112	0.97
23	Belgium	106	0.91

### 3.3. Active authors

**Figure 4.**
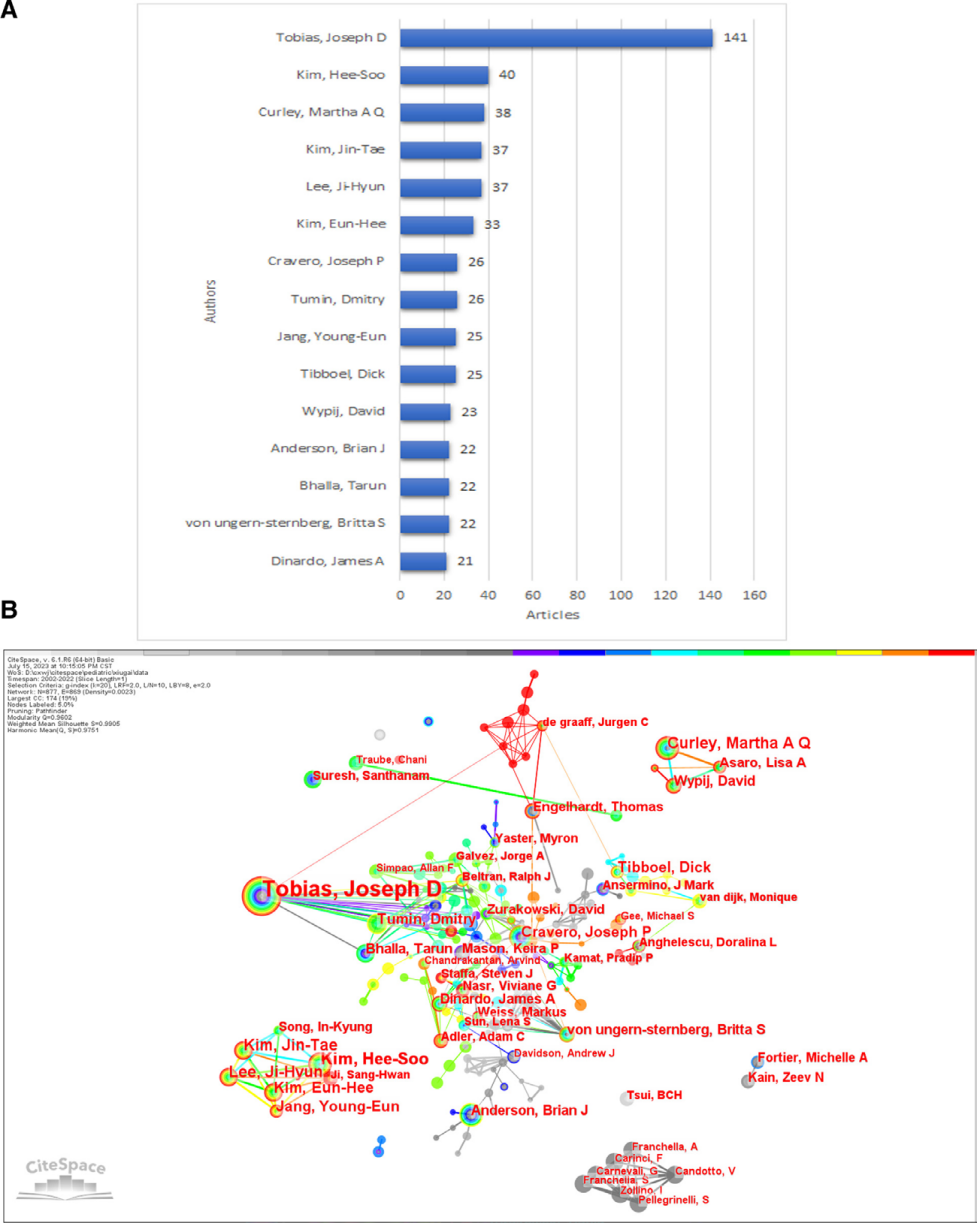
Analysis of author on pediatric anesthesia. (A) The rank of authors published more than 20 articles on pediatric anesthesia. (B) Network visualization map of author on pediatric anesthesia. (Citespace.6.1. R 6).

Of these articles, 30 authors output more than 12 articles (Table 2). Tobias, Joseph D topped the list with 141 articles published during this study and was far ahead of Kim, Hee-Soo (40) and Curley, Martha A Q (38), who were tied for second and third place respectively. Tobias, Joseph D worked at the Ohio State University, while Kim, Jin-Tae and Lee, Ji-Hyun worked at Seoul National University. The bar chart Figure 4A showed the top 15 active authors and the author occurrence diagram was showed in Figure 4B. From the picture, Candotto, V and his team output works in the early years. Kim, Jin-Tae, Lee, Ji-Hyun and Kim, Hee-Soo have been active and closely cooperated in this field in later years. De graaff, Jurgen C and his cooperators productive articles in recent years. Tobias, Joseph D leaded a big research team and cooperated with many teams. The network also showed other closely collaborating research teams like Curley, Martha A Q, von ungern-sternberg, Britta S and Fortier, Michelle A, ect.

**Table 2:** The active authors with more than 12 articles from 2002 to 2022 on pediatric anesthesia.

Ranking	Authors	Institution	Articles
1	Tobias, Joseph D	Ohio State University	141
2	Kim, Hee-Soo	Seoul Natl University	40
3	Curley, Martha A Q	Boston Childrens Hospital	38
4	Lee, Ji-Hyun	Seoul Natl University	37
5	Kim, Jin-Tae	Seoul Natl University	37
6	Kim, Eun-Hee	Seoul Natl University	33
7	Tumin, Dmitry	University N Carolina	26
8	Cravero, Joseph P	Boston Children’s Hospital	26
9	Tibboel, Dick	Erasmus MC-Sophia Children’s Hospital	25
10	Jang, Young-Eun	Seoul Natl University	25
11	Wypij, David	Harvard Medical School	23
12	Von ungern-sternberg, Britta S	University Western Australia	22
13	Bhalla, Tarun	Akron Children’s Hospital	22
14	Anderson, Brian J	University Auckland	22
15	Dinardo, James A	Boston Children’s Hospital	21
16	Mason, Keira P	Boston Children’s Hospital	19
17	Zurakowski, David	Boston Children’s Hospital	18
18	Engelhardt, Thomas	McGill University	17
19	Asaro, Lisa A	Boston Children’s Hospital	17
20	Weiss, Markus	Children’s Hospital Zürich	15
21	Song, In-Kyung	Seoul Natl University	15
22	Nasr, Viviane G	Boston Children’s Hospital	15
23	Kain, Zeev N	University Calif Irvine	15
24	Fortier, Michelle A	University Calif Irvine	15
25	Suresh, Santhanam	Ann and Robert H Lurie Childrens Hospital Chicago	14
26	Adler, Adam C	Texas Childrens Hospital	14
27	Yaster, Myron	Children’s Hospital Colorado	13
28	Staffa, Steven J	Boston Children’s Hospital	13
29	Ansermino, J Mark	BC Childrens Hospital	13
30	Anghelescu, Doralina L	St Jude Childrens Hospital	13

### 3.4. Productive institutions

Institution analysis result showed that Boston Childrens Hospital is the most productive affiliation, followed by Childrens Hospital Philadelphia and Ohio State University. The top 30 institutions output articles were shown in Table 3.

**Table 3:** Most productive institutions on pediatric anesthesia.

Institutions	RC	Institutions	RC
Boston Childrens Hospital	216	Cincinnati Childrens Hospital Med Ctr	116
Childrens Hospital Philadelphia	214	Johns Hopkins University	110
Ohio State University	214	University Michigan	102
University Penn	209	Emory University	99
Nationwide Childrens Hospital	204	University Calif San Francisco	91
Harvard Medical School	204	Seattle Childrens Hospital	88
University Toronto	194	Royal Childrens Hospital	86
University Washington	193	Mayo Clin	84
Harvard University	182	University Pittsburgh	83
Stanford University	162	Vanderbilt University	81
University Colorado	156	Duke University	80
Texas Childrens Hospital	143	Baylor Coll Med	77
Hospital Sick Children	139	University Cincinnati	76
Childrens Hospital	137	Childrens Hospital Colorado	74
North Western University	124	Yale University	72

RC = record count.

### 3.5. Citation analysis

**Figure 5.**
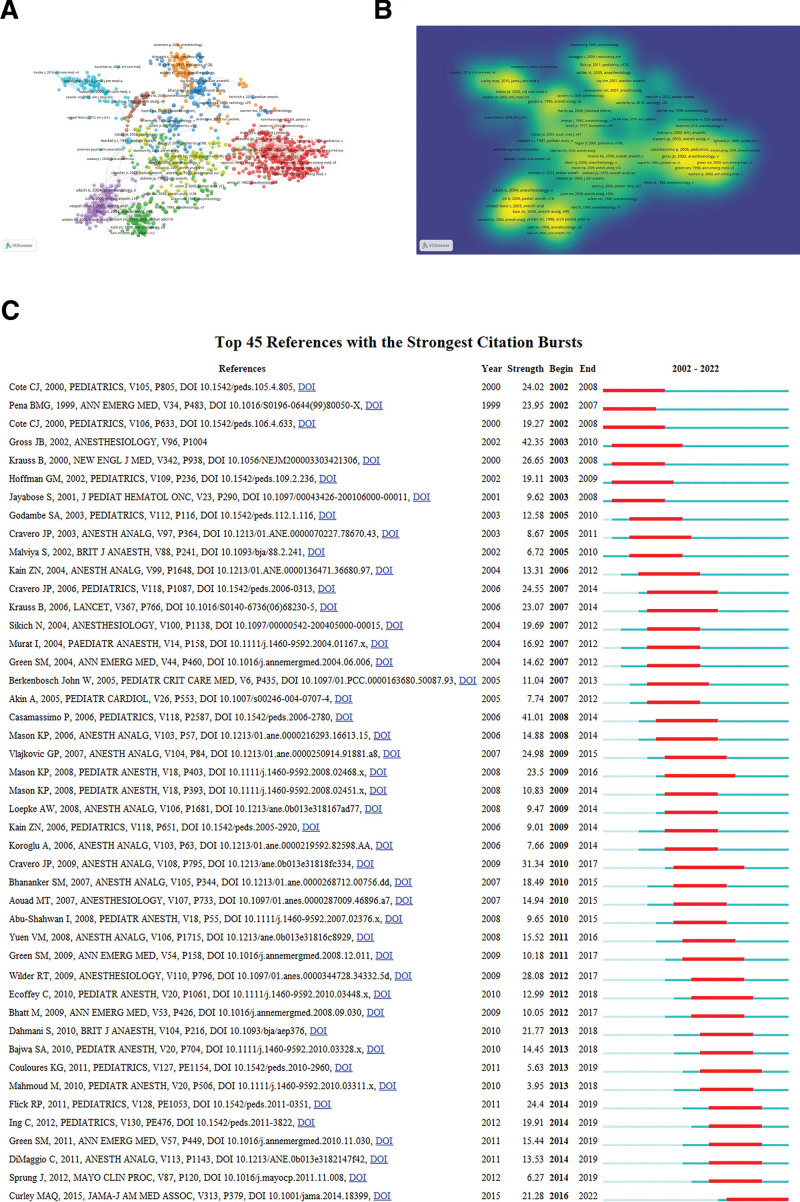
Reference analysis on pediatric anesthesia. (A) The reference network visualization on pediatric anesthesia. (VOSviewer version 1.6.16). (B) The density map of reference cluster on pediatric anesthesia. (C) The top 45 references with the strongest citation bursts. (Citespace.6.1. R 6).

The 11,591 articles cited 173,890 references. According to total citation numbers, there were 3 with more than 100 citations and 21 with more than 50 citations in our selected data. They are, in order, Habre W (2017) (Number of citations: 113), Gross JB (2002) (104), Cravero JP (2009) (100), Casamassimo P (2006) (98), Curley MAQ (2015) (97), Davidson AJ (2016) (96), Sun LS (2016) (93), Polaner DM (2012) (81), Flick RP (2011) (77), Wilder RT (2009) (72), Harris J (2016) (71), Mason KP (2008) (69), Traube C (2014) (67), Cravero JP (2006) (67), Krauss B (2006) (63), Ing C (2012) (63), Cote CJ (2016) (63), Vlajkovic GP (2007) (62), Dahmani S (2010) (60), Krauss B (2000) (55), and Cote CJ (2000) (51). We showed the details of the top 10 cited papers in Table 4.^[22–31]^ Network visualization and the density map were displayed in Figure 5A and B. And when we choose the minimum duration of 6 to search for strongest citation bursts, 45 burst articles have been found and displayed in Figure 5C.

**Table 4:** The top 10 cited articles on pediatric anesthesia.

No.	Title	Author	Journal	PY	Freq	DOI
1	Incidence of severe critical events in pediatric anesthesia (APRICOT): a prospective multicentre observational study in 261 hospitals in Europe	Habre W	*Lancet Resp Med*	2017	113	10.1016/S2213-2600 (17)30116-9
2	Practice Guidelines for Sedation and Analgesia by Non-Anesthesiologists	Gross JB	*Anesthesiology*	2002	104	10.1097/00000542-200204000-00031
3	The incidence and nature of adverse events during pediatric sedation/anesthesia with propofol for procedures outside the operating room: a report from the pediatric sedation research consortium	Cravero JP	*Anesth Analg*	2009	100	10.1213/ane.0b013e31818fc334
4	Guidelines for monitoring and management of pediatric patients during and after sedation for diagnostic and therapeutic procedures: an update	Casamassimo P	*Pediatrics*	2006	98	10.1542/peds.2006-2780
5	Protocolized sedation vs usual care in pediatric patients mechanically ventilated for acute respiratory failure: a randomized clinical trial	Curley MAQ	*Jama-J Am Med Assoc*	2015	97	10.1001/jama.2014.18399
6	Neurodevelopmental outcome at 2 years of age after general anesthesia and awake-regional anesthesia in infancy (GAS): an international multicentre, randomized controlled trial	Davidson AJ	*Lancet*	2016	96	10.1016/S0140-6736 (15)00608-X
7	Association between a single general anesthesia exposure before age 36 months and neurocognitive outcomes in later childhood	Sun LS	*Jama-J Am Med Assoc*	2016	93	10.1001/jama.2016.6967
8	Pediatric regional anesthesia network (PRAN): a multi-institutional study of the use and incidence of complications of pediatric regional anesthesia	Polaner DM	*Anesth Analg*	2012	81	10.1213/ANE.0b013e31825d9f4b
9	Cognitive and behavioral outcomes after early exposure to anesthesia and surgery	Flick RP	*Pediatrics*	2011	77	10.1542/peds.2011-0351
10	Early exposure to anesthesia and learning disabilities in a population-based birth cohort	Wilder RT	*Anesthesiology*	2009	72	10.1097/01.anes.0000344728.34332.5d

PY = publication year.

### 3.6. Most cited journals

**Figure 6.**
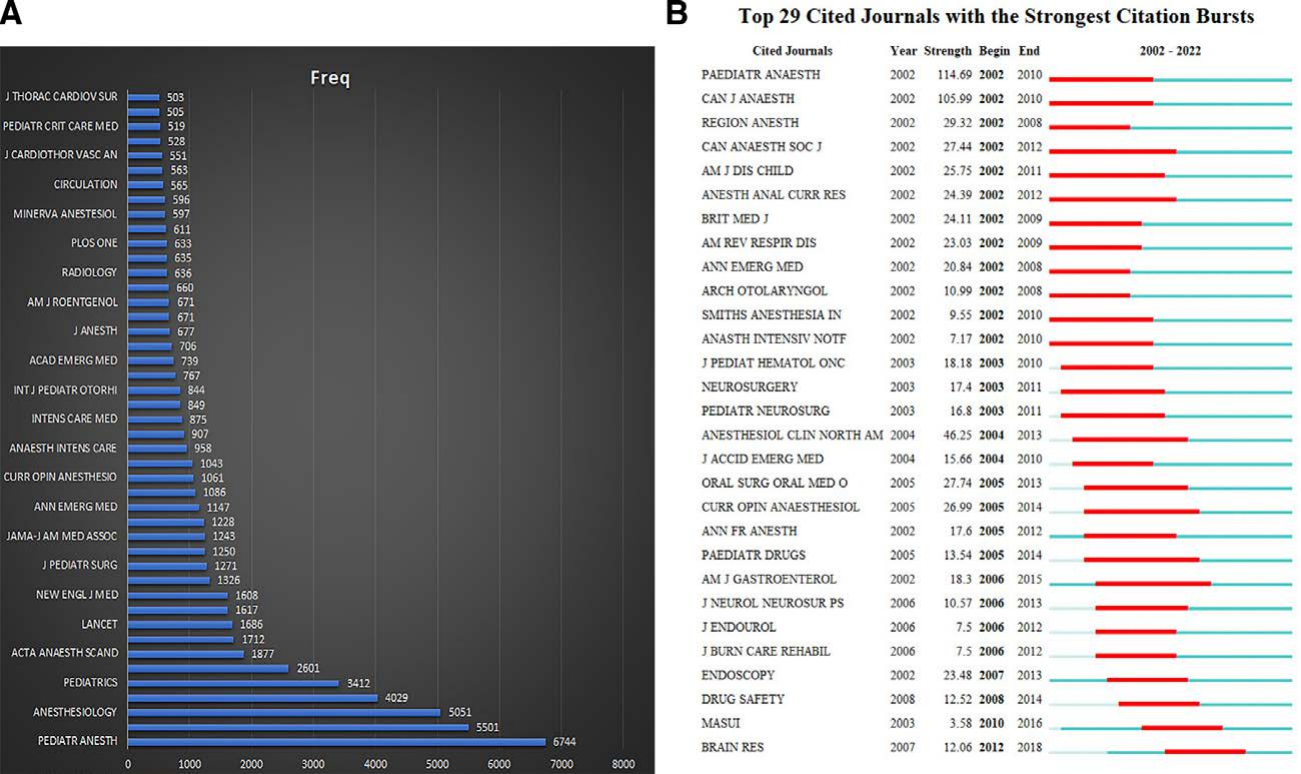
The most cited journals on pediatric anesthesia. (A) Cluster bar chart of journals with more than 500 cited frequent on pediatric anesthesia. (B) The top 29 cited journals with strongest citation burst (Citespace.6.1. R 6).

Among these articles and their references, the list of cited journals was statistically analyzed (Fig. 6). The top 5 cited journals were *Pediatric Anesth* (6744), *Anesth Analg* (5501), *Anesthesiology* (5051), *Brit J Anaesth* (4029 and *Pediatrics* [3412]). Forty-six journals cited by authors more than 500 times and the ranking showed in Figure 6A. Table 5 demonstrated the details of top 10 cited journals. The top 29 cited journals with strongest citation burst were shown in Figure 6B.

**Table 5:** The top 10 cited journals from 2002 to 2022 on pediatric anesthesia.

Rank	Journal name	Citation	IF (2023)	Quartile in category (2023)	Country	Cycle	OA
1	*Pediatr Anesth*	6744	1.7	Q3	United States	Monthly	No
2	*Anesth Analg*	5501	5.7	Q1	United States	Monthly	No
3	*Anesthesiology*	5051	8.8	Q1	United States	Monthly	No
4	*Brit J Anaesth*	4029	9.8	Q1	England	Monthly	No
5	*Pediatrics*	3412	8.0	Q1	United States	Monthly	No
6	*Anaesthesia*	2601	10.7	Q1	England	Monthly	No
7	*Acta Anaesth Scand*	1877	2.1	Q4	Denmark	Monthly	No
8	*Can J Anaesth*	1712	4.2	Q1	Canada	Bimonthly	No
9	*Lancet*	1686	168.9	Q1	England	Weekly	No
10	*J Clin Anesth*	1617	6.7	Q1	United States	Bimonthly	No

### 3.7. Keywords and trend topics

**Figure 7.**
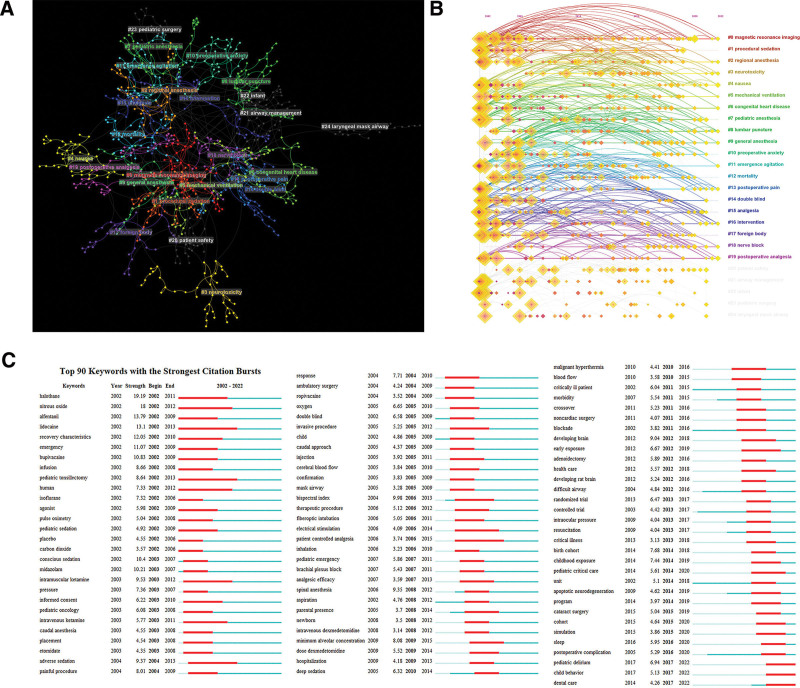
Keyword analysis on pediatric anesthesia. (A) The keywords network visualization on pediatric anesthesia. (B) The timespan map of keyword cluster on pediatric anesthesia. Each horizontal row is a cluster. Longitudinally, the node corresponds to the first appearance time of the keywords. (C) The top 90 keywords with the strongest citation bursts, this is a reprinted image and the videos of its analysis results are available in the supplemental content. (Citespace.6.1. R 6).

In the 11,591 articles, a total of 11,585 different keywords were found. When we set scale factor k = 25 and selected top 50 keywords from each year using citespace, 907 keywords were included in analysis. These keywords were divided into 25 clusters with different colors, and the network visualization map as shown in Figure 7A. The largest cluster is the cluster 0 (red), with 59 keywords, including magnetic resonance imaging, MRI, CT, event, therapy, brain, etc. Followed by the cluster 1 (orange), with 44 keywords like management, quality, care, risk factor, etc. Cluster 2 (deep orange) has 42 keywords, mainly including regional anesthesia, lidocaine, ropivacaine, caudal block, local anesthetics, etc. Cluster 3 (lemon yellow) has 41 keywords, containing neurotoxicity, disorder, developing brain, brain injury, bispectral index monitor, etc. Next, we analyzed the keywords timespan shown in Figure 7B. Then, the top 90 keywords with the strongest citation bursts on pediatric anesthesia were extracted and shown in Figure 6C. In addition, the most used medication in articles published about the topic of pediatric anesthesia were propofol (584), midazolam (393), sevoflurane (277), etc (Table 6).

**Table 6:** The top medicine from 2002 to 2022 on pediatric anesthesia.

Ranking	Medicine	Freq
1	Propofol	584
2	Midazolam	393
3	Sevoflurane	277
4	Ketamine	242
5	Fentanyl	193
6	Halothane	190
7	Dexmedetomidine	176
8	Nitrous oxide	169
9	Bupivacaine	158
10	Isoflurane	130
11	Remifentanil	123
12	Morphine	114

## 4. Discussion

According to our study (Fig. 2), a compelling increasing trend was found for the number of articles on the topic of pediatric anesthesia. The number of articles has increased from 202 in 2002 to 954 in 2022. Over 500 articles published annually in this field since 2012. Regression analysis were used to evaluate the trend of the annual articles published in this field, finding the increasing trend will persist. This result revel that researchers pay more and more attention on pediatric anesthesia in the world. It may further promote the development of pediatric anesthesiology.

When we investigate the productive countries or regions, found there were 23 countries or area output more than 100 articles (Fig. 3 and Table 1), including 18 developed areas (the USA, Canada, Germany, the United Kingdom, Italy, France, Japan, South Korea, Australia, Netherlands, Switzerland, Israel, Spain, Sweden, Austria, Belgium,) and 8 developing area (China, Turkey, India, Brazil, Egypt, Iran, Saudi Arabia). Among them, the number of articles in the top 5 are the United States, Canada, Turkey, China, India. It shows that the academic publication productivity is closely related to the size of economies and the level of national development. In addition, the top thirty active institutions showed in Table 3 were located in prosperous areas. It further shows that economic prosperity can greatly promote the output of academic works. Our study is consistent with the results of other bibliometric research in the literature on the relationship between national economic development level and the number of academic publications.^[9,32,33]^ If the author wants to choose an outstanding institution or affiliation in this field for further study, he may pay attention to these institutions in developed country like Boston Childrens Hospital, Childrens Hospital Philadelphia, Ohio State University, University Penn, Nationwide Childrens Hospital and so on.

When authorship was analyzed (Fig. 4 and Table 2), the authors including Tobias, Joseph D; Kim, Jin-TaeLee; Ji-Hyun; Martha A Q; Kim, Hee-Soo; Kim, Eun-Hee; Tibboel, Dick; Jang, Young-Eun; Tumin, Dmitry; Bhalla, Tarun, and Cravero, Joseph P have more than 20 articles in pediatric anesthesia. The results according to analysis of the author network map found the authors Tobias, Joseph D cooperated with many authors, joined more than 100 researches and output some outstanding studies from past to now.^[34–37]^ Moreover, authors Kim, Hee-Soo, Kim, Jin-Tae, and Lee, Ji-Hyun cooperated closely and produced some outstanding articles in this field in recent years.^[38–40]^ As an old Chinese saying goes, one who stays near vermilion gets stained red. Researchers could read their articles or join outstanding teams to comprehensively learn and understand children’s anesthesia-related research.

When the literature was evaluated according to the total number of citations (Fig. 4 and Table 4), Habre W et al^[22]^ study published in the journal of *Lancet Respiratory Medicine* in 2017 ranked first in our study interval with 187 citations. Then Gross JB’s paper titled “Practice guidelines for sedation and analgesia by non-anesthesiologists” published in Anesthesiology in 2002 won the second place.^[26]^ The third is the paper about the rescue processing for propofol adverse during pediatric sedation published in the journal of *Anesth Analg* in 2009 by Cravero JP.^[27]^ When we set the minimum duration as 6 to find the Burstness citations, 45 articles were filtered. Article published in the journal of *Anesthesiology* in 2002 by Gross JB got the strongest citation burst with 42.35 strength from 2003 to 2010. After this research, with the most citation burst was the study published in the journal of *Pediatrics* in 2006 by Casamassimo P. The third study still from author Gravero JP (2009).^[41]^ It is suggested that people who interested in this topic may first read these articles mentioned in this review. Also, we analyzed the most cited journals. *And Anesth Analg, Anesthesiology, Pediatr Anesth, Brit J Anaesth, Pediatrics* were our highly recommended authoritative journals (Fig. 6 and Table 5). Recently, *Japanese Journal of Anesthesiology and Brain Research* are also good choice.

The research frontiers could be determined by analyzing the frequency of keywords.^[42,43]^ Therefore, we analyzed the keywords of the literature and found that there are a total of 11,585 keywords in these articles, of which 98 keywords appeared more than 100 times. Except for words similar to children or pediatrics, the top 10 keywords with high degree centrality and frequency of occurrence are nitrous oxide, CT, congenital heart disease, risk factor, pharmacokinetics, fentanyl, bispectral index, MRI, delirium, pediatric surgery. In these keywords, we may know researchers pay more attention to different aided examinations and pharmacokinetics to help reducing the risk of pediatric anesthesia. Then the top 907 keywords were selected to generate a network map as shown in Figure 7. According to timeline analysis results, the time span of cluster0, cluster 2, cluster 4, cluster 5, cluster 6, cluster 8, cluster 9, cluster 11, cluster 13, cluster 16 and cluster 19 were from 2002 to 2022. Cluster 20 to 24 including fewer keywords from 2002 to 2021. From the strongest citation burst keywords (Fig. 7C, the videos of its analysis results are available in the supplemental content, http://links.lww.com/MD/K291), halothane, nitrous oxide, alfentanil, lidocaine, recovery characteristics were the focus of research in early years. Newborn, intravenous dexmedetomidine, minimum alveolar concentration, deep sedation and others were the focus in middle years. In recent years, the topics trends were determined to be cohort, simulation, sleep, postoperative complication, pediatric delirium, child behavior, and dental care have been becoming the significant focus of researchers attention. Beginners can find research inspiration and breakthrough points according to these hot spots.

This study also has limitations. First, the data were collected from only 8 databases commonly used in WoS. Although most researchers believe that WoS is a better choice for bibliometric analysis, some articles may be missed.^[44–46]^ Secondly, a large variety of publications were published in English, which may lead to selection bias in terms of writing language. Third, our research topic is too extensive, which is not conducive to researchers to explore the application of different anesthesia and sedation methods in children.

## 5. Conclusion

In conclusion, study on the topic of pediatric anesthesia have obtained growing attention. The noticeable increase in the number of annual publications indicates that this research field has gained importance worldwide. Turkey ranked third in the number of publications, after the United States and Canada. This study has identified the key researchers and institutions involved in children anesthesia related research globally. Tobias, Joseph D is the most active researchers while the Boston Childrens Hospital was the most productive institution in this research field. The most popular journal in this field is *Anesth Analg*. Pediatric delirium, child behavior and dental care are considered to be the hotspots now, while safe and comfortable medical care for children may be the focus in the future. These findings help new researchers quickly understand the authoritative institutions, authors, literature and frontier trends in this field and further provide with an exhaustive outlook about pediatric anesthesia.

## Author contributions

**Conceptualization:** Dijiao Ruan, Jing Hua.

**Formal analysis:** Lianlian Li.

**Methodology:** Xiaoli Li.

**Project administration:** Jing Hua, Xiaoli Li.

**Software:** Dijiao Ruan, Xiaoli Li, Lianlian Li.

**Supervision:** Jing Hua.

**Validation:** Lianlian Li.

**Visualization:** Dijiao Ruan, Lianlian Li.

**Writing – original draft:** Dijiao Ruan, Xu Tang.

**Writing – review & editing:** Dijiao Ruan, Jing Hua, Xu Tang.

## Supplementary Material


